# Supplementary material for: Antigenicity of the Mu (B.1.621) and A.2.5 SARS-CoV-2 Spikes
Source: Viruses. 2022 Jan 14;14(1):144. doi: 10.3390/v14010144 (PMC8780535; doi:10.3390/v14010144)
Supplement: Supplementary file 1 [file viruses-14-00144-s001.zip › viruses-1529941-supplementary.pdf]

viruses

MDPI

Supplementary Materials

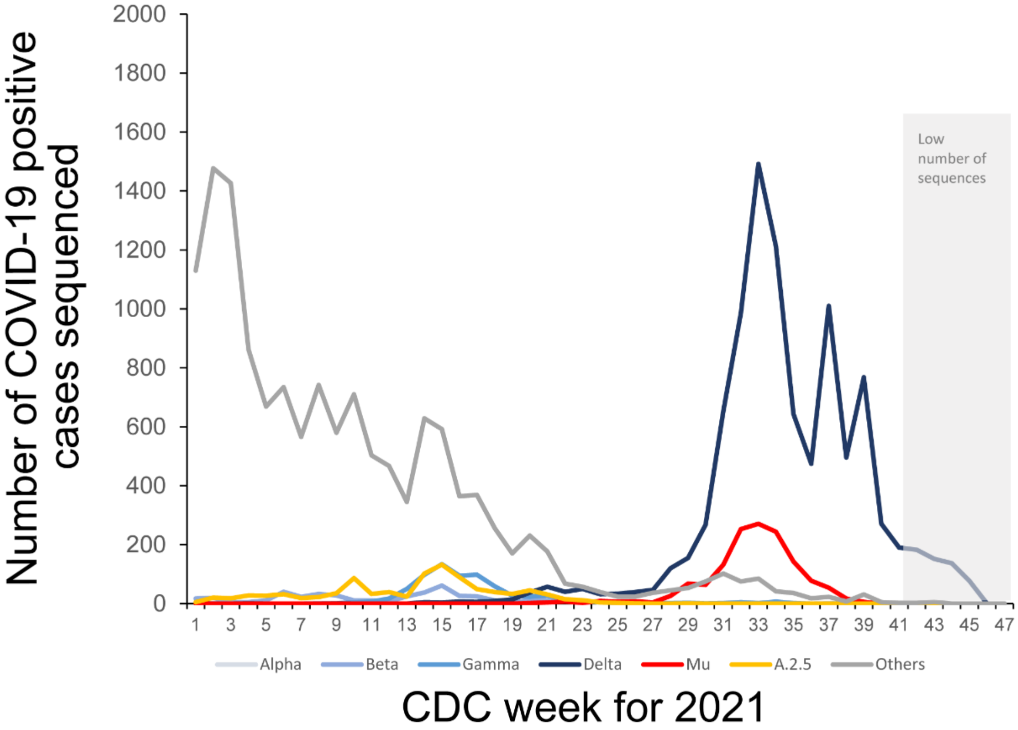

Figure S1. Distribution of COVID-19 positive cases sequenced by CDC week for variants Alpha, Beta, Gamma, Delta, Mu, A.2.5 and others. The Mu variant was present in Québec from CDC week 27 (4th of July) to CDC week 42 (17th of October). The A.2.5 variant was present at low level from the beginning of 2021 to CDC week 29 (18th of July).

Table S1. Binding Kinetics of the interaction between SARS-CoV-2 RBD and sACE2 quantified by Biolayer Interferometry.

**Ligands**	**Temperature**	ACE2 binding (RBD/biolayer interferometry)		
		**KD (nM)**	**Ka (1/Ms)**	**Kdis (1/s)**
**WT**	10°C	57.19	$2.68x10^4$	$1.53x10^{-3}$
	25°C	162.6	$4.72x10^4$	$7.68x10^{-3}$
**R346K**	10°C	45.71	$2.67x10^4$	$1.22x10^{-3}$
	25°C	166.4	$4.64x10^4$	$7.72x10^{-3}$
**K417N**	10°C	120	$5.09x10^4$	$6.11x10^{-3}$
	25°C	264.2	$6.45x10^4$	$1.70x10^{-2}$
**L452R**	10°C	68.17	$1.91x10^4$	$1.30x10^{-3}$
	25°C	148.3	$2.98x10^4$	$4.41x10^{-3}$
**E484K**	10°C	56.39	$2.99x10^4$	$1.69x10^{-3}$
	25°C	146.8	$5.58x10^4$	$8.18x10^{-3}$
**N501Y**	10°C	11.47	$2.91x10^4$	$3.33x10^4$
	25°C	35.45	$4.42x10^4$	$1.57x10^{-3}$

Table S2. Summary of ACE2 Binding, plasma binding, and neutralization to variants.

SARS CoV-2 Variants	ACE2 binding[a]	Spike recognition by plasma[b]		Neutralization (ID_{50})[c]	
		**Naïve vaccinated** (3 week post vaccination of 2nd dose)	**Previously infected vaccinated** (3 week post vaccination of 2nd dose	**Naïve vaccinated** (3 week post vaccination of 2nd dose)	**Previously infected vaccinated** (3 week post vaccination of 2nd dose)
D614G	1	1	1	1186.8 (1)	2668.2 (1)
B.1.351 (**Beta**)	4.1	0.70	0.84	190.4 (0.16)	744.4 (0.27)
B.1.617.2 (**Delta**)	2.32	0.58	0.58	325 (0.27)	374.2 (0.14)
B.1.621 (**Mu**)	1.69	0.66	0.63	145.5 (0.12)	343.4 (0.12)
A.2.5	2.04	0.72	0.71	361.3 (0.30)	905 (0.33)

a) ACE2-Fc binding was normalized to CV3-25 binding in each experiment. Values are presented as ratio of normalized ACE2-Fc binding obtain with the D614G Spike. Values represent the means of data obtained from at least three independent experiments.

b) Plasma binding were normalized to CV3-25 in each experiment. Values are presented as ratio of normalized plasma binding obtained with the D614G Spike. Values represent the means of data obtained with 9-10 plasma from the same group.

c) The ID_{50} represents the plasma dilution to inhibit 50% of the infection of 293T-ACE2 cells by pseudo viruses bearing the indicated Spike. Values are presented as the means of ID_{50} and in parenthesis as ratio of the ID_{50} obtained with virus bearing the D614G Spike. Values represent the means of data obtained with 9-10 different plasma.
